# Production of multiple bacteriocins, including the novel bacteriocin gassericin M, by *Lactobacillus gasseri* LM19, a strain isolated from human milk

**DOI:** 10.1007/s00253-020-10493-3

**Published:** 2020-03-13

**Authors:** Enriqueta Garcia-Gutierrez, Paula M. O’Connor, Ian J. Colquhoun, Natalia M. Vior, Juan Miguel Rodríguez, Melinda J. Mayer, Paul D. Cotter, Arjan Narbad

**Affiliations:** 1 Gut Microbes and Health Institute Strategic Programme, Quadram Institute Bioscience, Norwich, UK; 2 Food Bioscience Department Teagasc Food Research Centre, Moorepark, Fermoy, Cork, Ireland; 3 APC Microbiome Ireland, University College Cork, Cork, Ireland; 4 Molecular Microbiology, John Innes Centre, Norwich, UK; 5 Dpt. Nutrition and Food Science, Complutense University of Madrid, Madrid, Spain

**Keywords:** Bacteriocin, Antimicrobial, SCFA, Gassericin, Colon model, *Lactobacillus gasseri*

## Abstract

Bacteriocins are antimicrobial peptides produced by bacteria, and their production is regarded as a desirable probiotic trait. We found that *Lactobacillus gasseri* LM19, a strain isolated from human milk, produces several bacteriocins, including a novel bacteriocin, gassericin M. These bacteriocins were purified from culture and synthesised to investigate their activity and potential synergy. *L. gasseri* LM19 was tested in a complex environment mimicking human colon conditions; it not only survived, but expressed the seven bacteriocin genes and produced short-chain fatty acids. Metagenomic analysis of these in vitro colon cultures showed that co-inoculation of *L. gasseri* LM19 with *Clostridium perfringens* gave 16S ribosomal RNA metagenomic profiles with more similarity to controls than to vessels inoculated with *C. perfringens* alone. These results indicate that *L. gasseri* LM19 could be an interesting candidate for maintaining homeostasis in the gut environment.

**Electronic supplementary material:**

The online version of this article (10.1007/s00253-020-10493-3) contains supplementary material, which is available to authorized users.

## Introduction

Beneficial bacteria have consistently been harnessed throughout human history. Most recently, the rise of antimicrobial resistance among pathogens, a greater demand for healthy foods and an increasing appreciation of the importance of the human gut microbiota have brought attention back to natural sources of new antimicrobials, food preservatives and probiotics. The search for natural antimicrobials can involve a variety of approaches (Lewis 2013), including taking advantage of the fact that bacteria from a specific environmental niche are able to compete against other bacteria from the same niche (Czárán et al. 2002; Kelsic et al. 2015). Such bacterial antagonism can be through non-specific strategies, like the production of organic acids. Some organic acids, particularly the short-chain fatty acids (SCFA), acetate, propionate and butyrate, are produced in millimolar quantities in the gastrointestinal (GI) tracts of animals and humans and, in addition to their antagonistic activities, confer other health benefits (LeBlanc et al. 2017; Singh et al. 2018). Target-specific antagonistic activities can be provided by compounds such as bacteriocins (Garcia-Gutierrez et al. 2018), a heterogeneous group of ribosomally synthesised peptides. They represent a potential alternative to traditional antibiotics because of their frequent low toxicity, high potency, ability to be bioengineered, low likelihood of resistance development and the possibility of being produced in situ by probiotics (Cotter et al. 2013; Field et al. 2015; Hegarty et al. 2016).

*Lactobacillus* spp. are members of the lactic acid bacteria (LAB) and contribute to the production of many fermented foods, as well as being important components of the human gut microbiota and sources of antimicrobial peptides (Collins et al. 2017). *Lactobacillus gasseri* is one of six species which previously comprised the *Lactobacillus acidophilus* complex (Fujisawa et al. 1992; Sarmiento-Rubiano et al. 2010). These species are considered ecologically and commercially important and have been extensively studied, frequently revealing antimicrobial and other probiotic properties (Abramov et al. 2014; Karska-Wysocki et al. 2010; Kim et al. 2007; Selle and Klaenhammer 2013; Yamano et al. 2007). *L. gasseri* strains have been isolated from the gut of animals and humans, vaginal tract, human milk and oral cavity and have been found to produce different classes of bacteriocins, frequently referred to as gassericins. Gassericin A is a cyclic class IIc bacteriocin produced by *L. gasseri* LA39, from an infant faecal sample (Kawai et al. 1994; Pandey et al. 2013). Gassericins B1, B2, B3 and B4 were isolated from vaginal isolate *L. gasseri* JCM 2124, with B1 and B3 being identical to the α and β peptides of the two-component bacteriocin acidocin J1132 from *L. acidophilus* (Tahara et al. 1997). Production of gassericin T (GasT) was first reported in *L. gasseri* SBT 2055, a strain isolated from adult human faeces (Kawai et al. 2000). Although bacteriocins of class IIb have previously been shown to have one active peptide and one inactive complementary factor, the second peptide from the same cluster, GatX, was also shown to have antimicrobial activity (Mavrič et al. 2014). Acidocins LF221A and LF221B were isolated from *L. acidophilus* LF221 (later renamed *L. gasseri* LF221), from infant faeces (Bogovic-Matijasic et al. 1998). *L. gasseri* K7 was also isolated from the faeces of a breast-fed baby and two two-peptide bacteriocin-encoding operons were found in its genome (Zorič Peternel et al. 2010). These potential peptides showed high homology to acidocins LF221A and LF221B and gassericin T peptides, respectively (Mavrič et al. 2014). The gassericin E operon was identified in *L. gasseri* EV1461, isolated from the vagina of a healthy woman; it encodes both antimicrobial gassericin E, with high similarity to gassericin T, and gene *gaeX*, whose product is identical to GatX and gassericin K7 B (Maldonado-Barragán et al. 2016). Recently, genes encoding gassericin T (GatA and GatX) and the novel gassericin S, with similarity to acidocin LF221A (GasA and GasX), were found in the genome of *L. gasseri* LA327, isolated from the colon of a human adult (Kasuga et al. 2019). This study demonstrated synergistic activity between the two components of gassericin T, and those of gassericin S, but not between gassericin S and gassericin T (Kasuga et al. 2019).

In this study, we aimed to characterise the antimicrobial and probiotic potential of a novel strain, *L. gasseri* LM19, isolated from human milk. We found that *L. gasseri* LM19 exhibits antimicrobial activity against different enteropathogens and possesses three bacteriocin clusters in its genome, including one encoding a novel bacteriocin, designated gassericin M. We examined antimicrobial activity and synergy using both purified and synthesised peptides. In order to examine potential probiotic use, we also demonstrated that *L. gasseri* LM19 survives, expresses all the bacteriocin genes, and produces SCFA in detectable amounts in a complex faecal environment mimicking colon conditions. In addition, it can help to maintain the composition of the microbiome in the presence of the pathogen *Clostridium perfringens*.

## Methods

### Isolation and whole genome sequencing of *L. gasseri* LM19

*L. gasseri* LM19 was originally isolated from breast milk on MRS agar (Oxoid España SA, Madrid, Spain) at 37 °C and has been deposited in the National Collection of Industrial, Food and Marine Bacteria (NCIMB, Aberdeen, UK) with the accession number NCIMB 15251. Whole genome sequence was provided by MicrobesNG (Birmingham, UK) using Illumina® HiSeq and a 250 bp paired end protocol. Genome coverage was × 30. Reads were trimmed using Trimmomatic 0.30 with a sliding window quality cutoff of Q15 (Bolger et al. 2014), and the quality was assessed using software Samtools (Li et al. 2009), BedTools (Quinlan and Hall 2010), and BWA mem (Li and Durbin 2010). SPAdes 3.7 (Bankevich et al. 2012) was used for de novo assembly, and annotation was performed using Prokka 1.11 (Seemann 2014).

### Bioassay-based screening for antimicrobial activity

Antimicrobial overlay assays, cross streaks, drop tests, filter disc tests and well diffusion assays were performed as described previously (Garcia-Gutierrez 2019), supplementing plates with 2% *w*/*v* NaHCO_3_ to counteract inhibition from lactic acid. Well diffusion assays incorporated 100 μl of an overnight culture of the incubator strains. Bacterial strains used were from culture collections (ATCC, American Type Culture Collection, Manassas, Virginia, USA; DSMZ, Deutsche Sammlung von Mikroorganismen und Zellkulturen, Braunschweig, Germany; NCTC, National Collection of Type Cultures, London, UK) or in-house collections. Strains and culture conditions were: *L. gasseri* LM19 (MRS, 37 °C, anaerobic, static), *Salmonella enterica* LT2 (LB, 37 °C, anaerobic, static), *Escherichia coli* ATCC 25922 (LB, 37 °C, anaerobic, static), *Cronobacter sakazakii* DSMZ 4485 (BHI, 37 °C, anaerobic, static), *C. perfringens* NCTC 3110 (BHI, 37 °C, anaerobic, static), *Listeria innocua* NCTC 11288 (BHI, 37 °C, anaerobic, static), *Lactobacillus delbrueckii* subsp. *bulgaricus* (*L. bulgaricus*) 5583 (MRS, 37 °C, anaerobic, static), *L. bulgaricus* LMG 6901 (MRS, 37 °C, aerobic, static), *Campylobacter jejuni* NCTC 11168 (Brucella medium, 37 °C, microaerobic, static), and *Micrococcus luteus* FI10640 (MRS, 37 °C, aerobic, static). Media was sourced from Oxoid (Hampshire, UK).

### In silico identification of bacteriocin gene clusters

The *L. gasseri* LM19 genome was analysed with software to identify putative bacteriocin clusters: BAGEL 3 and BAGEL 4 (van Heel et al. 2013) to target bacteriocin clusters and antiSMASH to target secondary metabolites (Weber et al. 2015). Genome data was visualised using Artemis (Carver et al. 2012). DNA and amino acid sequences identified as putative bacteriocin genes and proteins were analysed using BLAST (Altschul et al. 1990) using default parameters. Geneious Tree Builder v11.1 (Biomatters Ltd., Auckland, New Zealand) was used to compare the gassericins.

### Detection and purification of antimicrobial peptides

*L. gasseri* LM19 was grown anaerobically at 37 °C in 2 l MRS broth for 24–48 h. The culture was centrifuged (8000×*g*, 20 min, 10 °C), and cells and supernatant were analysed independently. The cell pellet was resuspended in 400 ml IPA (70% propan-2-ol, 0.1% trifluoroacetic acid) for 3–4 h at room temperature, centrifuged again and the supernatant retained for further purification. This sample was tested for antimicrobial activity using *L. bulgaricus* LMG 6901 as an indicator and was also analysed directly by matrix assisted laser deionisation-time of flight-mass spectrometry (MALDI-TOF-MS; Axima TOF^2^ MALDI-TOF mass spectrometer in positive-ion reflectron mode, Shimadzu Biotech, Manchester, UK) to determine the masses of the potential peptides. For further purification, IPA was removed by rotary evaporation until the sample volume was 120 ml; this was applied to a 2 g 12 ml Strata® C_18_-E solid-phase extraction (C18-SPE) column (Phenomenex, Macclesfield, Cheshire, UK), pre-equilibrated with methanol and water following manufacturer’s instructions. The column was washed with 20 ml 30% ethanol, then 20 ml 30% acetonitrile and the active fraction was eluted with 30 ml IPA. The IPA was removed as before and 4 ml aliquots were applied to a semi preparative Jupiter C5 Reversed Phase HPLC column (10 × 250 mm, 10 μm, 300 Å, Phenomenex) (“HPLC fractionation I”) running a 30–70% acetonitrile, 0.1% formic acid (FA) gradient over 95 min where buffer A is 0.1% FA and buffer B is 100% acetonitrile, 0.1% FA. Flow rate was 2.5 ml/min, and fractions were collected at 1-min intervals and further analysed by MALDI-TOF-MS.

For purification from the cell-free supernatant, the supernatant was applied to an Econo-column (BioRad, Watford, UK) containing 60 g Amberlite XAD 16 N. The column was washed with 400 ml 35% ethanol followed by 400 ml 30% acetonitrile and antimicrobial activity eluted with 450 ml IPA. IPA was removed by rotary evaporation until the sample volume was 145 ml; this was applied to a 5 g 20 ml C18-SPE column pre-equilibrated with methanol and water. The column was washed with 30 ml 30% ethanol followed by 30 ml 30% acetonitrile and antimicrobial activity eluted with 30 ml IPA and fractionated by semi-preparative reversed phase HPLC as before. To increase purity, some HPLC fractions were reapplied to the C5 semi prep column, running shallower gradients (“HPLC fractionation II”) (30–40% acetonitrile, 0.1% FA gradient over 95 min for GamX and Bact_2, 30–45% gradient for GamA, and 35–65% gradient for Bact_1, GamM and GamY).

Additionally, the six peptides were synthesised using microwave-assisted solid phase peptide synthesis on a Liberty Blue microwave peptide synthesiser (CEM Corporation, Charlotte, NC, USA). GamA and GamM were synthesised on H-Lys (BOC)-HMPB-ChemMatrix® resin, GamX was synthesised on H-Asn(Trt)-HMPB-ChemMatrix® resin, Bact_1 and Bact_2 on H-Arg(PBF)-HMPB-ChemMatrix® resin and GamY on Fmoc-Phe-Wang (Novobiochem®, Darmstadt, Germany) resin. Crude peptide was purified using RP-HPLC on a Semi Preparative Vydac C4 (10 × 250 mm, 5 μ, 300 Å) column (Grace, Chicago, Illinois, USA) running acetonitrile, 0.1% trifluoroacetic acid gradients specific to the peptide of interest. Fractions containing the desired molecular mass were identified using MALDI-TOF-MS and were pooled and lyophilised on a Genevac HT 4X lyophiliser (Genevac Ltd., Ipswich, UK). All naturally produced peptides and synthetic peptides were assayed by well-diffusion assay using *L. bulgaricus* DPC6901.

### Fermentation studies

*L. gasseri* LM19 was inoculated at 1% in 20 ml of prepared in-house MRS without glucose (10 g/l trypticase peptone (Difco, Wokingham, UK), 2.5 g/l yeast extract (Difco), 3 g/l K_2_HPO_4_, 3 g/l KH_2_PO_4_, 2 g/l tri-ammonium citrate, 0.2 g/l pyruvic acid, 0.3 g/l cysteine-HCl, 0.575 g/l MgSO_4_^.^7H_2_O, 0.12 g/l MnSO_4_^.^7H_2_O, 0.034 g/l FeSO_4_ 7H_2_O and 1 ml Tween 80 (all from Sigma-Aldrich, Gillingham, UK)), or batch model media, prepared as described previously (Parmanand et al. 2019). The pH was adjusted to 6.8 in both media and filter sterilised carbohydrate (glucose, lactose, galactose, inulin, starch or pectin [Sigma]) was added at 2% after autoclaving. Fermentations were incubated in anaerobic conditions at 37 °C over 48 h, conducted in triplicate and 2 ml samples collected at 24 h and 48 h. One millilitre was used for enumeration by plate count, pH measurement using a pH-000-85282 probe (Unisense, Aarhus, Denmark) and, once filter sterilised, well diffusion assay and 1 ml for RNA extraction.

### Nucleic acid extraction and sequencing

Samples for RNA extraction were mixed with two volumes of RNAlater (Sigma-Aldrich), centrifuged for 10 min at 18,000×*g* at 4 °C and pellets stored at − 80 °C. Extraction was performed using the RNeasy extraction kit (Qiagen, Manchester UK) with minor modifications. Pellets were resuspended in 1 ml RLT buffer, supplemented with 10 μl β-mercaptoethanol (Millipore) and transferred to lysing matrix E tubes (MP Biomedicals, Loughborough, UK). Samples were lysed in a FastPrep-24 homogeniser (MP Biomedicals) by applying 2 pulses of 30 s and intensity 6.0 with 1 min on ice between pulses. Samples were centrifuged for 10 min at 17,000×*g* and the supernatant transferred to clean 15 ml tubes and mixed with an equal volume of 70% ethanol. Seventy percent of the mixture, including any precipitate, was transferred to spin tubes and centrifuged at 8000×*g* for 1 min and following steps were as the manufacturer’s instructions. RNA was eluted in 100 μl RNase-free water and quantified by NanoDrop 2000 (Thermo Scientific, Gloucester, UK). DNase treatment was performed using the Turbo DNA-free™ kit (Invitrogen, Inchinnan, UK).

Genomic DNA was extracted using genomic tip-20 and genomic buffer set kits (Qiagen). Metagenomic DNA was extracted using the FastDNA Spin Kit for Soil (MP Biomedicals) following manufacturer’s guidelines. Total DNA concentration was measured by Qubit 3 (Invitrogen). The V4 region of the 16S rRNA gene was used for high-throughput sequencing using the Illumina MiSeq platform. Data analysis was conducted using Quantitative Insights into Microbial Ecology (QIIME2 version 2018.11) (Bokulich et al. 2018).

### Quantitative PCR (qPCR)

qPCR was used to detect the presence or absence of *C. perfringens* (Nagpal et al. 2015) and *L. gasseri* LM19 bacteriocin genes (Treven et al. 2013). Primers (Table 1, Sigma Genosys, Haverhill, UK) were designed using Primer 3 (v. 0.4.0) (Untergasser et al. 2012) and Netprimer (Premier Biosoft, San Francisco, California, USA) and tested using genomic DNA. Thermal cycling was performed using a Verity 96 well Thermal Cycler (Applied Biosystems, Cheshire, UK) using GoTaq G2 DNA polymerase (Promega, Chilworth, UK) according to manufacturer’s instructions. Reaction conditions were 20 s at 95 °C, 40 cycles of 1 s at 95 °C, 20 s at 60 °C and 15 s at 95 °C, and a melt curve of 15 min at 65 °C. dNTPs were provided by Bioline (London, UK). PCR products were visualised using 2% agarose gels. A standard curve for *C. perfringens* NCTC 3110 was constructed by extracting gDNA as described previously (Ladero et al. 2011) from a culture with known colony-forming units (cfu)/ml of *C. perfringens* NCTC 3110 then performing serial dilutions. Each DNA concentration was measured using qPCR to determine the cycle signal associated with each cell concentration.

**Table 1:** Primers for qPCR and RT-qPCR studies in *L. gasseri* LM19 and detection of *C. perfringens*

Gene	Accession number	Primer	Sequence (5′–3′)	Product size	Reference/accession no.
*gyrase A L. gasseri* LM19	7791_LM19_00854	LGgyrAF	TTGATTGCCTTAACCCTTCG	136	This work
		LGgyrAR	TTCCCATTGAACGAACATCA		
*bact_1*	7791_LM19_00792	Cluster 1.1F	TATTGGTGCATGGAGAGGTG	124	This work
		Cluster 1.1R	CCAGCCCACACATTGTACTG		
*bact_2*	7791_LM19_00793	Cluster1.2F	TTGGGGTAGTGTTGCAGGAT	97	This work
		Cluster1.2R	TGATGTTGCAGCTCCGTTAG		
*helveticin J-like*	7791_LM19_00025	Cluster2F	CTTGGGTACAAAGCGGAGAA	176	This work
		Cluster2R	GCCTGCTCGGTTAAGATAAG		
*gamA* (=*gasT*)	7791_LM19_00116	Cluster3.1F	CTGGATGGGCTCTTGGAAAT	112	This work
		Cluster3.1R	TTTCCGAATCCACCAGTAGC		
*gamX* (=*gatX*)	7791_LM19_00117	Cluster3.2F	TGGGGGAATGCTGTAATAGG	100	This work
		Cluster3.2R	CTCCTAAGCCACAGGCAGTC		
*gamY*	7791_LM19_00122	GamYF	ACTCAAATCGTAGGAGGAAAAGG	150	This work
		GamYR	AAAGCATGCACCTGAACCA		
*gamM*	7791_LM19_00123	GasMF	AGCAGGAGGAGCATTTTCAA	90	This work
		GasMR	CCTGCTGCACCACCTAAAAT		
Immunity gene *gamI3*	7791_LM19_00118	Cluster 3.3F	CAGATGAAGAAGCATTACTTGAAAA	102	This work
		Cluster 3.3R	TTCCAGGCCAAGTATTAGTTGTA		
*C. perfringens 16S*	MN960263	s-Clper-F	GGGGGTTTCAACACCTCC	170	(Nagpal et al. 2015)
		ClPER-R	GCAAGGGATGTCAAGTGT		

### Quantitative reverse transcription PCR (RT-qPCR)

cDNA synthesis was carried out using the QuantiTect® Reverse Transcription Kit (Qiagen, UK) using 100 ng RNA per reaction. RT-qPCR was performed using 384-well plates (4titude Ltd., Dorking, UK) in the ViiA™ 7 System (Applied Biosystems) with the SensiFAST™ SYBR® No-ROX Kit (Bioline, London, UK). Reaction mix composition was, in 6 μl, 0.6 μl of cDNA template, 3 μl of 2x SensiFAST SYBR® No-ROX mix, 0.24 μl of each primer (10 μM stock) and 1.92 μl of water. Reaction conditions were as qPCR. Reactions were set up in duplicate and the baseline for change was 2-fold relative to housekeeping gene *gyrase A.*

### Transformation of *L. gasseri* LM19

Electro-competent cells of *L. gasseri* LM19 were made based on the method described previously (Holo and Nes 1989). Competent cells were resuspended in 2.25 ml 10% glycerol/0.5 M sucrose, aliquoted in volumes of 40 μl and either used immediately or frozen on dry ice. Five hundred nanograms of plasmid pUK200 (Wegmann et al. 1999) were added to 40 μl of electro-competent cells. The mixture was incubated for 1 min on ice and transferred to a pre-chilled electroporation cuvette (Geneflow Limited, Lichfield, UK). A pulse of 1500 V, 800 Ω and 25 μF was applied using a BioRad electroporator (Watford, UK). Four hundred fifty microlitres of pre-chilled MRS/ 20 mM MgCl_2_/2 mM CaCl_2_ were added to the cuvette, and the mixture transferred to a chilled 2-ml tube and incubated for 2 h at 37 °C. Aliquots were plated on MRS with 7.5 μg/ml chloramphenicol and incubated overnight at 37 °C. Transformants were confirmed by colony PCR using Go Taq G2 polymerase and primers p181 (5′-GCGAAGATAACAGTGACTCTA-3′ and p54 (5′-CGGCTCTGATTAAATTCTGAAG-3′).

### In vitro colonic batch model fermentation

Fermentations to simulate human colon conditions were performed as described previously (Parmanand et al. 2019). Experiments were carried out using three different faecal donors, one fermentation for each donor. Each faecal fermentation comprised 4 vessels—control, inoculation with *L. gasseri*, inoculation with *C. perfringens*, co-inoculation with both *L. gasseri* and *C. perfringens.* Overnight cultures of *L. gasseri* LM19 pUK200 and *C. perfringens* NCTC 3110 were added to the vessels at 1% each. Six-millilitre samples were extracted at 0 h, 4 h, 8 h, 24 h and 48 h for DNA and RNA extractions, SCFA analysis and, from those vessels where *L. gasseri* had been added alone, enumeration of *L. gasseri* LM19 pUK200 by plate count on MRS supplemented with 7.5 μg/ml chloramphenicol.

### SCFA analysis

SCFA were measured using proton NMR (Parmanand et al. 2019). The metabolites were quantified using Chenomx NMR Suite v8.12 (Chenomx Inc., Edmonton, Canada) with the TSP concentration set to 0.1 mM.

### Statistical analysis

Significant differences between groups were established using a paired *t* test, assuming normal distribution, equal variances. Both sides of the distribution were considered. Significance was considered when the *p* value was < 0.05.

## Results

### Antimicrobial activity

The antimicrobial activity of *L. gasseri* LM19 was assessed against Gram-positive and Gram-negative pathogens (Table 2). The assay method affected the outcome, with the targets typically being more sensitive to LM19 grown on agar than to its cell free supernatant. The growth of *C. perfringens* NCTC 3110 and *L. bulgaricus* 5583 was inhibited by overlay assays. Cross-streaks showed antimicrobial activity against all Gram-positive indicators and *C. sakazakii*, while supernatants exhibited activity by filter disc against *L. bulgaricus*, *C. jejuni* and *M. luteus*. Well-diffusion assay only inhibited the growth of *L. bulgaricus*. As *L. bulgaricus* was the most sensitive indicator, it was used in subsequent tests.

**Table 2:** Summary of inhibitory activity of *L. gasseri* LM19 using different techniques

	Overlay	Cross-streak	Drop test	Filter disc	Well-diffusion
*S. enterica* LT2	–	–	–	–	–
*E. coli* ATCC 25922	–	–	–	–	–
*C. sakazakii* NCTC 11467	–	++	–	–	–
*C. perfringens* NCTC 3110	+	+	–	–	–
*L. innocua* NCTC 11288	–	++	–	–	–
*L. bulgaricus* 5583	+++	+++	–	++	+++
*C. jejuni* NCTC 11168	np	np	np	+	np
*M. luteus* FI10640	–	++	–	+	–

−, no activity; + 1 mm radius inhibition zone; ++, 1–5 mm radius inhibition zone; +++, > 5 mm inhibition zone; np, not performed

### Identification of bacteriocin gene clusters in the genome of *L. gasseri* LM19

The sequenced genome of *L. gasseri* LM19 was assembled into contigs and submitted to the NCBI under accession number SHO00000000. AntiSMASH 3.0 indicated the presence of a single Microcin M-like cluster, while BAGEL 4, which specifically targets regions with bacteriocin similarities, found three clusters predicted to encode a number of potential bacteriocins. Manual investigation confirmed the presence of two clusters, whose putative structural peptides showed a high similarity to antimicrobial peptides from class IIb bacteriocins (clusters 1 and 3), and a helveticin-like protein (cluster 2). The latter contained no other bacteriocin-associated genes on the basis of Blastp analysis; the product of the single gene showed 31.9% identity and 43.1% amino acid consensus to helveticin J, which was originally characterised in *Lactobacillus helveticus* following heterologous expression (Joerger and Klaenhammer 1986).

**Fig.1:**
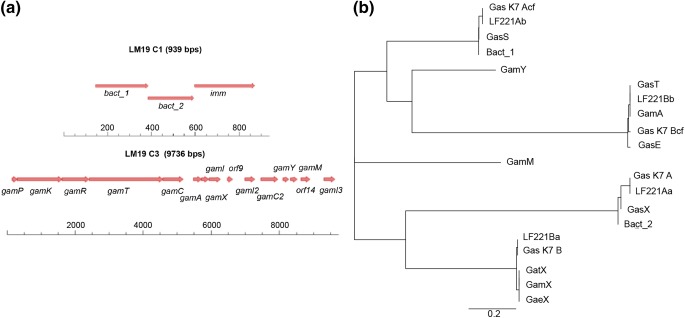
**a** Gene organisation in *L. gasseri* LM19 predicted bacteriocin cluster 1 (LM19 C1), encoding Bact_1 and Bact_2 and an immunity gene (imm), and cluster 3 (LM19 C3), encoding GamA, GamX, GamY and GamM and accessory genes. **b** Phylogenetic tree of the amino acid sequences of putative bacteriocins identified in *L. gasseri* LM19 in context with other class IIb gassericins

Cluster 1 (939 bp) has 99% nucleotide identity to the class IIb gassericin K7A cluster (EF392861). The cluster was predicted to encode two short peptides with leader sequences (Bact_1 and Bact_2) and a putative immunity protein (Fig. 1a). Bact_1 and Bact_2 show 100% amino acid similarity with the gassericin S structural peptides GasA and GasX respectively (Kasuga et al. 2019), while the surrounding genes do not resemble any other genes associated with bacteriocin production. The putative immunity protein showed 97% amino acid homology to those of the acidocin LF221A and gassericin S clusters (Kasuga et al. 2019; Majhenič et al. 2004).

Cluster 3 is 9736 bp and the first open reading frames (orfs) 1–8 show a high nucleotide homology to the gassericin T cluster from *L. gasseri* LA158 (AB710328, 99% over 100% coverage) and the gassericin E cluster from EV1461 (KR08485, 99% over 95% coverage) (Fig. 1a). There are two structural peptide-encoding genes, *gamA* and *gamX*, that are preceded by homologues of the gassericin E cluster as described previously (Maldonado-Barragán et al. 2016). It is likely that they perform the same predicted functions as their gassericin E homologues, i.e. *gamP*, *gamK*, *gamR* for regulation, *gamT* and *gamC* for transport and, after the structural peptides, *gamI* for immunity, although a homologue to *gaeX* is missing. The predicted GamA peptide has the same sequence as GasT, Gas K7B cf. and acidocin LF221B cf. and has a single amino acid difference (W-L) from GasE (Table 3). The second putative peptide, GamX, has the same sequence as GatX and GaeX, all of which differ by a single amino acid (G-A) from Gas K7 B and acidocin LF221B (Table 3).

**Table 3:** Bacteriocins described in *L. gasseri*

Gassericin	Amino acid sequence	Molecular mass (Da)	Reference
A	**MIEKVSKNELSRIYGG**NNVNWGSVAGSCGKGAVMGIYFGNPILGCANGAATSLVLQTASGIYKNYQKKR	5652	(Kawai et al. 1994; Pandey et al. 2013)
B1	(N-terminal) NPKVAHCASQIGRSTAWGAVSGAATGTAVGQAVGA-X	6217	(Tahara et al. 1997)
B2	(N-terminal) MISKPEKNTLRL-X	4400	(Tahara et al. 1997)
B3	(N-terminal) GNPKVAHCASQIGRSTAW-X	6273	(Tahara et al. 1997)
B4	(N-terminal) NPKVAHCASQIGRSTAW-X	5829	(Tahara et al. 1997)
GatX	**MALKTLEKHELRNVMGG**NKWGNAVIGAATGATRGVSWCRGFGPWGMTACGLGGAAIGGYLGYKSN	4763	(Mavrič et al. 2014)
Acidocin LF221A α	**MIEKVSKNELSRIYGG**NNVNWGSVAGSCGKGAVMEIYFGNPILGCANGAATSLVLQTASGIYKNYQKKR	3393	(Bogovic-Matijasic et al. 1998)
Acidocin LF221A β(cf)	**MKVLNECQLQTVVGG**KNWSVAKCGGTIGTNIAIGAWRGARAGSFFGQPVSVGTGALIGASAGAIGGSVQCVGWLAGGGR	5523	(Bogovic-Matijasic et al. 1998)
Acidocin LF221B α	**MALKTLEKHELRNVMGG**NKWGNAVIGAATGATRGVSWCRGFGPWGMTACALGGAAIGGYLGYKSN	3393	(Bogovic-Matijasic et al. 1998)
Acidocin LF221B β	**MKNFNTLSFETLANIVGG**RNNWAANIGGVGGATVAGWALGNAVCGPACGFVGAHYVPIAWAGVTAATGGFGKIRK	5542	(Bogovic-Matijasic et al. 1998)
K7 A	**MIEKVSKNELSRIYGG**NNVNWGSVAGSCGKGAVMEIYFGNPILGCANGAATSLVLQTASGIYKNYQKKR	5523	(Zorič Peternel et al. 2010)
K7 A (cf)	**MKVLNECQLQTVVGG**KNWSVAKCGGTIGTNIAIGAWRGARAGSFFGQPVSVGTGALIGASAGAIGGSVQCVGWLAGGGR	3393	(Zorič Peternel et al. 2010)
K7 B	**MALKTLEKHELRNVMGG**NKWGNAVIGAATGATRGVSWCRGFGPWGMTACALGGAAIGGYLGYKSN	4777	(Zorič Peternel et al. 2010)
K7 B (cf)	**MKNFNTLSFETLANIVGG**RNNWAANIGGAGGATVAGWALGNAVCGPACGFVGAHYVPIAWAGVTAATGGFGKIRK	5542	(Zorič Peternel et al. 2010)
GasE	**MKNFNTLSFETLANIVGG**RNNLAANIGGVGGATVAGWALGNAVCGPACGFVGAHYVPIAWAGVTAATGGFGKIRK	5469	(Maldonado-Barragán et al. 2016)
GamA (=GasT)	**MKNFNTLSFETLANIVGG**RNNWAANIGGVGGATVAGWALGNAVCGPACGFVGAHYVPIAWAGVTAATGGFGKIRK	5542	This work, (Kawai et al. 2000)
GamX (=GaeX)	**MALKTLEKHELRNVMGG**NKWGNAVIGAATGATRGVSWCRGFGPWGMTACGLGGAAIGGYLGYKSN	4763	This work
Bact_1 (=GasS)	**MKVLNECQLQTVVGG**KNWSVAKCGGTIGTNIAIGAWRGARAGSFFGQPVSVGAGALIGASAGAIGGSVQCVGWLAGGGR	6060	This work, (Kasuga et al. 2019)
Bact_2 (=GasX)	**MIEKVSKNELSRIYGG**NNVNWGSVAGSCGKGAVMGIYFGNPILGCANGAATSLVLQTASGIYKNYQKKR	5451	This work, (Kasuga et al. 2019)
GamY	**MKTLNEQELTQIVGG**KGNKGINWANVRCASAVTIGALGGGLAGPGGMVGGFLLGSGACF	4105	This work
GamM	**MRKINKEELVEITGG**FNAAKCAVGTAGGAFSIARGSAAFGVPGMVIGGILGGAAGALASCK	4124	This work

X, sequence not available; cf., complemental factor; leader sequences are marked in bold where known

In cluster 3, there are 7 further orfs including two additional putative structural genes, designated as *gamM* and *gamY*, which appear to encode a further two-component bacteriocin. These putative peptides also show some similarity to other two-peptide component gassericins, but to a lesser extent (Fig. 1b). GamY shows similarity to GamM, with 25.4% identity and 47.6% consensus, and they both have similarity to K7 A cf. (27.5% identity and 38.8% consensus; 25.3% identity, 44.3% consensus, respectively) and to GamA (18.7% identity, 33.3% consensus with GamM). Surrounding *gamY* and *gamM* are two genes encoding putative immunity proteins, GamI2 and GamI3, with homology to an enterocin A immunity domain (pfam 08951, 2.8e^−7^ and 1.1e^−6^, respectively), a putative transport accessory protein, GamC2, with some similarity to TIGR01295 bacteriocin transport accessory protein (1.18e^−9^), and thioredoxin superfamily cd02947 (5.21e^−7^), and two orfs with no matches. The genes on either side of the cluster show amino acid homology to transporters involved in solute or cation transport, and so are not predicted to be part of the cluster.

### Identification of antimicrobial peptides in culture

**Fig. 2:**
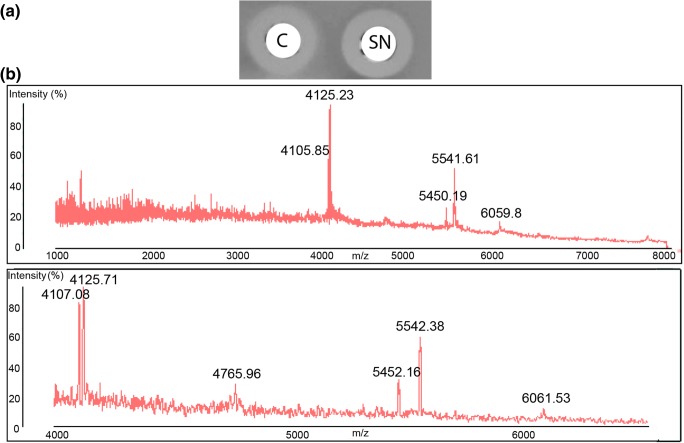
**a** Antimicrobial activity of cell (C) and supernatant (SN) IPA extracts of *L. gasseri* LM19 culture. **b** Mass spectra showing masses of interest for the six peptides in two different cell extracts

**Fig. 3:**
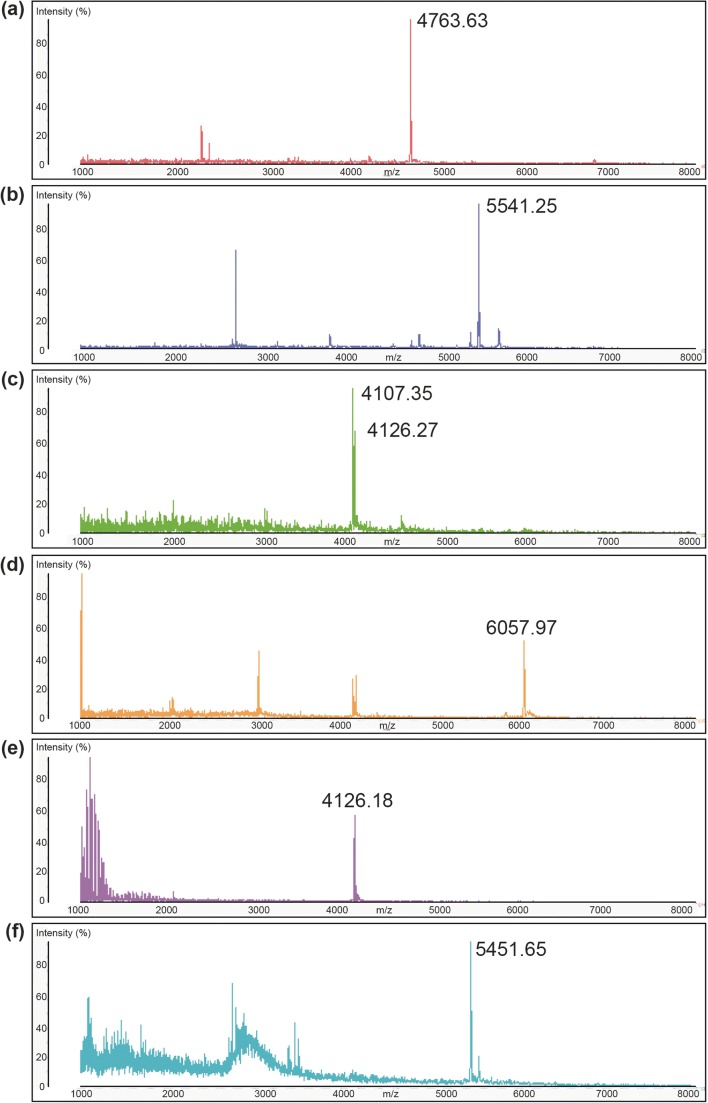
MS spectra of active fractions from HPLC-purified cell extracts (**a**–**e**) and supernatant (**f**). Cell extract samples purified by HPLC fractionation I showed putative masses for **a** GamX, 4763 Da; **b** GamA, 5541 Da; **c** GamM, 4126 Da and GamY, 4107 Da; **d** Bact_1, 6057 Da; **e** putative GamM, 4126 Da, was separated from GamY by HPLC fractionation II; **f** a mass for Bact_2, 5451 Da, was identified after HPLC fractionation I of culture supernatant

Extraction of both cells and culture supernatants with IPA demonstrated the presence of antimicrobial activity in both samples (Fig. 2a), while MALDI TOF MS analysis of cell extracts demonstrated the presence of peptide masses that were consistent with those predicted by in silico analysis for GamM (4125 Da), GamY (4105 Da), Gam A (5541 Da), Bact_2 (5450 Da), Bact_1 (6059 Da) and Gam X (4765 Da) (Table 3, Fig. 2b). Further purification of these cell extracts by HPLC and analysis of fractions showing antimicrobial activity allowed separation of many of these masses (Fig. 3), identifying masses corresponding to putative GamX (4763 Da, Fig. 3a), GamA, (5541 Da, Fig. 3b), co-eluted GamY and GamM (4107 and 4126 Da respectively, Fig. 3c) and Bact_1 (6057 Da, Fig. 3d), while further purification (HPLC fractionation II) allowed the separation of GamM (4126 Da, Fig. 3e).

MS analysis of culture supernatant samples from HPLC fractionation I which showed antimicrobial activity identified a mass for Bact_2 (5451 Da, Fig. 3f).

### Antimicrobial activity of fractions and synergy between synthetic peptides

**Fig.4:**
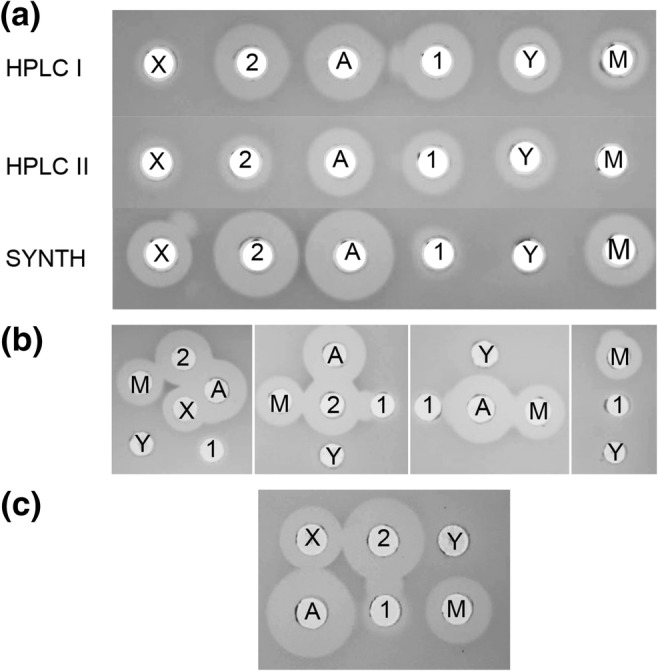
**a** Antimicrobial activity of fractions containing peptides of masses corresponding to GamX, (X); Bact_2, (2); GamA, (A); Bact_1, (1); GamY (Y); GamM, (M) from HPLC fractionation I (HPLC I), from cell extracts and supernatant, and from HPLC fractionation II (HPLC II) - MS chromatograms of these fractions are presented in Supplemental Fig. S3. Activity from HPLC fractionations I and II was compared with the activity of the synthetic peptides (SYNTH). **b** Synergy between the different synthetic peptides. **c** Synergy between pairs of synthetic peptides

The antimicrobial activities of three sets of fractions were compared, fractions from HPLC fractionation I of cell extracts (Supplemental Fig. S1), from HPLC fractionation II of these fractionation I samples (Supplemental Fig. S2), and synthetic peptides resuspended in milli Q water at 1 mg/ml. MS profiles of all samples demonstrate that fractions contained separated masses corresponding to all six peptides, although the level of GamX was very low from HPLC fractionation I and HPLC fractionation II gave very small yields of GamY and GamM (Supplemental Fig. S3). All synthetic peptides except GamY showed antimicrobial activity, with the highest activity coming from GamA and Bact_2 (Fig. 4a). Samples from HPLC fractionations also gave clear antimicrobial activity, with GamA and Bact_2 again giving the largest zones of inhibition, and here the fractions containing the mass for GamY showed clear activity (Fig. 4a). The low activity of GamX and, from the second fractionation, GamM, might be due to the low yields of the peptides, and variation in activity between fractionations I and II may also be due to differing yields in fractions (Supplemental Fig. S3). Figure 4b and c show synergy assays between synthetic peptides. We observed clear synergy between Bact_1 and Bact_2 and a possible synergy between Bact_1 and GamA. No synergy was observed between GamM and GamY or between GamA and GamX. Control tests confirmed that IPA did not affect bacterial growth (data not shown).

### Complex carbohydrates can favour viability and antimicrobial activity of *L. gasseri* LM19

**Fig. 5:**
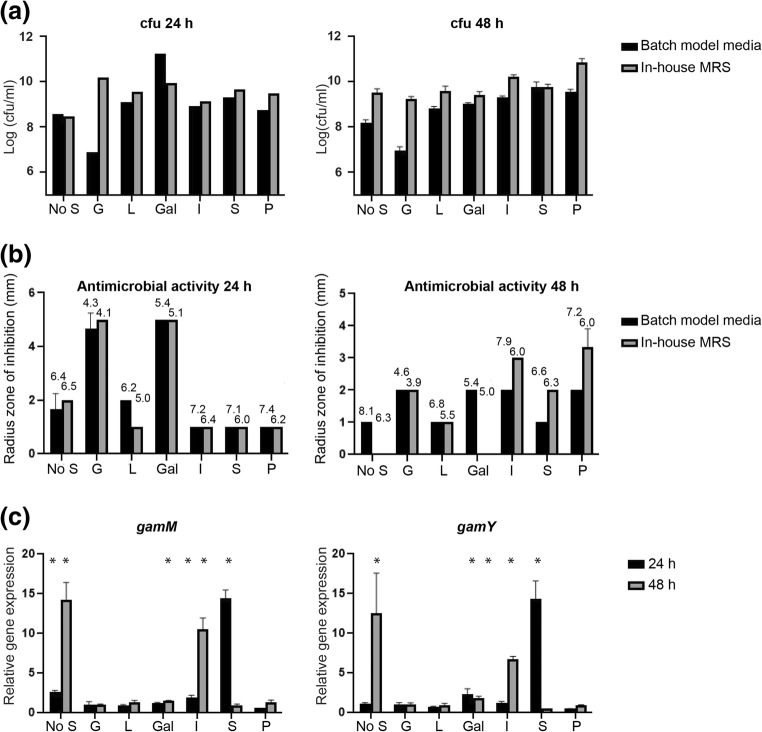
**a** Viable counts of *L. gasseri* LM19 recovered after growth in batch model media or home-made MRS supplemented with different carbon sources. **b** Antimicrobial activity of cultures in **a** measured by well diffusion assay (Figures above bars indicate mean pH). **c** Gene expression levels of *gamM* and *gamY* after *L. gasseri* LM19 was cultured in home-made MRS supplemented with different carbon sources. Expression values were normalised with those of housekeeping gene *gyrase A*. Values are relative to the gene expression measured in the glucose treatment, which was given the arbitrary value of 1. No S, no supplementation; G, glucose; L, lactose; Gal, galactose; I, inulin; S, starch and P, pectin; *, significant difference to glucose supplementation (*p* < 0.05). Results are the mean of triplicate measurements ± standard deviation

*L. gasseri* LM19 was grown in colon batch model medium, simulating gut conditions, or home-made MRS, alone or supplemented with simple sugars (glucose, lactose and galactose) or complex polymers (inulin, starch and pectin). In general, more viable cells were recovered from MRS; growth on simple sugars was highest at 24 h but, at 48 h, complex carbohydrates gave higher counts (Fig. 5a). Interestingly, growth in the absence of a carbon source at 48 h was similar to that with simple sugars. On batch model medium, cell counts with glucose were lower than with all other treatments, galactose produced the highest counts at 24 h while starch and pectin improved growth at 48 h. Antimicrobial activity from batch model medium with glucose was almost as high as that from MRS despite a ~ 3 log difference in cfu (Fig. 5b). Glucose and galactose supplementation showed the highest antimicrobial activity at 24 h, while complex carbohydrates inulin and pectin produced the highest activity after 48 h.

The changes in activity with carbon supplementation over time suggest control of antimicrobial production in different nutritional environments. Examination of bacteriocin gene expression by RT-qPCR in MRS also showed that an absence of carbon supplementation increased the expression of *gamM* and *gamY* significantly at 48 h. Starch supplementation increased the expression of both genes at 24 h, as did inulin at 48 h. Galactose supplementation also produced a significant increase in expression of *gamM* at 48 h and *gamY* at 24 and 48 h (Fig. 5c). Other bacteriocin genes did not show notable changes in expression, except for an increase in expression of the *helveticin J-like* gene in the presence of starch at 24 h (Supplementary Table S1).

### In vitro colon model fermentations with *L. gasseri* LM19

#### Survival of *L. gasseri* LM19 and *C. perfringens* in an in vitro colon model

*L. gasseri* LM19 was transformed with a plasmid conferring chloramphenicol resistance to allow selection and enumeration of this strain within a mixed microbial community. Transformation of electrocompetent cells gave an efficiency of 1.07 × 10^2^ transformants/ng of DNA. Fermentations with three different faecal donors were performed on three separate occasions with four vessels per fermentation inoculated with *L. gasseri* LM19-pUK200, *C. perfringens* NCTC 3110, *L. gasseri* with *C. perfringens*, or a media control. Viable *L. gasseri* counts were measured in the treatment vessel inoculated with *L. gasseri* LM19-pUK200 alone by selective plating. The numbers recovered increased from 5.30, 5.22 and 5.22 log_10_ cfu/ml in donors 1, 2 and 3, respectively at 4 h, to 6.12, 6.39 and 6.36 log_10_ cfu/ml at 8 h and 7.30, 7.31 and 7.47 log_10_ cfu/ml at 24 h. However, after 48 h, levels of recovery dropped to 3.66, 4.00 and 3.72 log_10_ cfu/ml. It was not possible to assess *L. gasseri* LM19 counts in the *C. perfringens* co-inoculated vessel, as *C. perfringens* was able to grow on the selective plates. However, analysis of *L. gasseri* LM19 housekeeping gene *gyrase A* mRNA levels by RT-qPCR showed that copy numbers were similar in vessels treated with either *L. gasseri* LM19 alone or with *L. gasseri* with *C. perfringens* (data not shown).

**Fig. 6:**
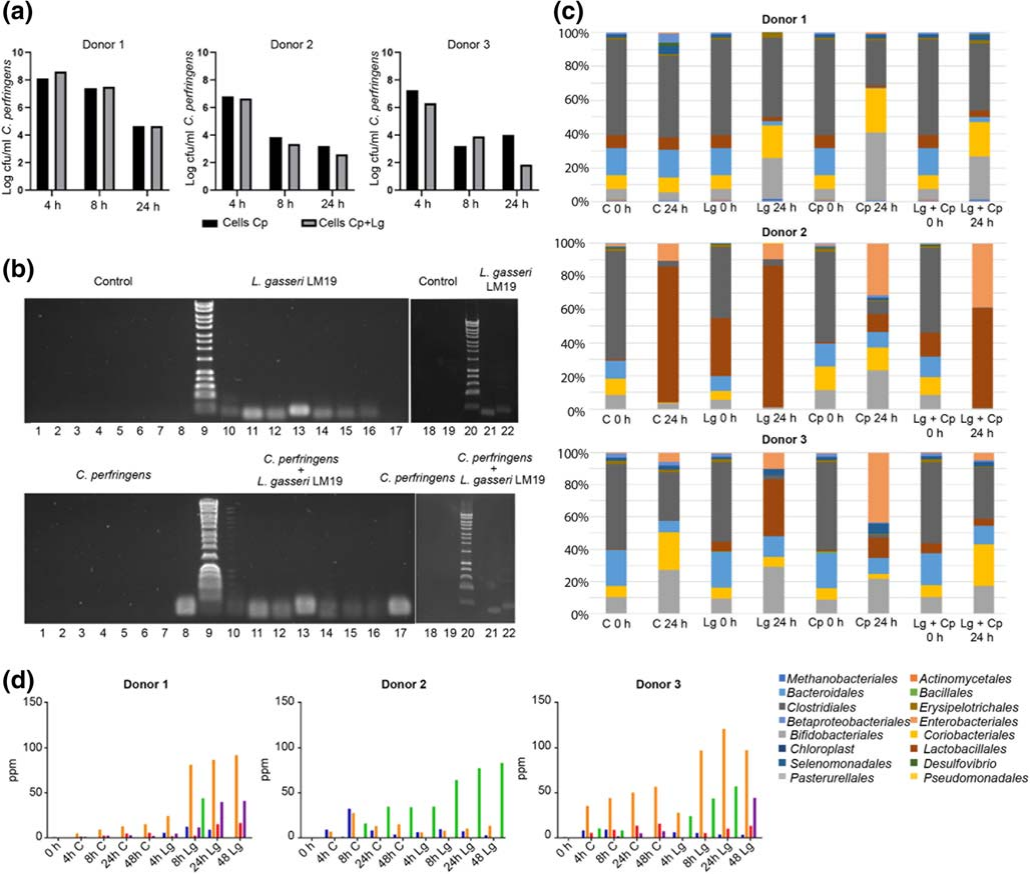
In vitro colon model fermentations with *L. gasseri* LM19 and *C. perfringens.***a***C. perfringens* NCTC 3110 population measured by qPCR when inoculated alone (Cp) or co-inoculated with *L. gasseri* LM19 (Cp + Lg) in three different fermentations with faeces from donors 1, 2 and 3. **b** Expression of bacteriocin genes in vessels from all 4 treatments (control, *L. gasseri* LM19 alone, *C. perfringens* alone, *L. gassieri* LM19 with *C. perfringens*) at 24 h in fermentations with faeces from donor 1: lane 1, negative control; lanes 2 and 10, *bact_1*; lanes 3 and 11, *bact_2*; lanes 4 and 12, *gamA*; lanes 5 and 13, *gamX*; lanes 6 and 14, *helveticin J-like*; lanes 7 and 15, *gyrA*; lanes 8 and 17, *C. perfringens*; lanes 9 and 20, molecular marker; lanes 18 and 21, *gamM*; lanes 19 and 22, *gamY*. **c** Microbial profiles obtained by 16S rRNA analysis showing relative abundance at the order level in the faecal batch model fermentation for the three donors (C, control, Lg, *L. gasseri* LM19 alone, Cp, *C. perfringens* alone, Lg + Cp, co-inoculation of *L. gasseri* LM19 and *C. perfringens*). **d** Production of SCFA in batch model faecal fermentations from control vessels (C) and vessels inoculated with *L. gasseri* LM19 alone (Lg) using faecal inoculum from three different donors: blue, formate; orange, acetate; red, propionate; purple, butyrate; green, lactate

*C. perfringens* levels were measured by qPCR, which detects DNA from both live and dead cells. Addition of *L. gasseri* LM19 did not have a negative effect on the *C. perfringens* population in the fermentation with faecal sample from donor 1; there was a tendency to lower *C. perfringens* counts in co-culture at 24 h with donors 2 and 3, but the changes were not significant (Fig. 6a).

#### Bacteriocin gene expression

RT-qPCR was used to detect expression of the bacteriocin genes *bact_1, bact_2, helveticin-J like*, *gamA*, *gamX*, *gamM* and *gamY*, compared to housekeeping gene *gyrase A*. This showed detectable levels of bacteriocin gene expression at 24 h both in the vessel where *L. gasseri* LM19 was inoculated alone and in co-culture with *C. perfringens* (Fig. 6b). Primers specific to *C. perfringens* only identified a signal in the two vessels where *C. perfringens* had been inoculated. At 48 h, expression of only *helveticin-J like*, *gamM* and *gamY* genes was detected (data not shown).

#### Impact of *L. gasseri* LM19 on gut microbiota composition

Changes in the colon model microbiota were analysed by 16S rRNA metagenomic profiling. Analysis of relative abundance at order, family and genus level was conducted. The initial bacterial composition was, as expected, different between the three donors (Fig. 6c). Bacterial populations from donor 1 remained relatively stable over 24 h. The addition of *L. gasseri* LM19, *C. perfringens* or both did not result in a significant increase in proportions of *Lactobacillales* or *Clostridiales*, and all 3 treatments resulted in similar increases in *Bifidobacteriales* and *Coriobacteriales* relative to the control, with the *L. gasseri* LM19 with *C. perfringens* co*-*treatment being more similar to the *L. gasseri* LM19 only condition.

The initial population from donor 2 was constituted mainly of *Clostridiales*, with some *Bacteroidales*, *Coriobacteriales* and *Bifidobacteriales*. A change can be observed at 24 h in both the control and the samples where *L. gasseri* LM19 or *L. gasseri* LM19 with *C. perfringens* were added, with an increase in *Lactobacillales* along with a small increase in *Enterobacteriales*. The decrease in relative abundance of *Bifidobacteriales* and *Coriobacteriales* in the control, *L. gasseri* and *C. perfringens* with *L. gasseri* treatments was statistically significant (*p* < 0.05) and not observed in the *C. perfringens* sample. *C. perfringens* alone appeared to prevent the overgrowth of *Lactobacillales*, while *Clostridiales* were decreased, being replaced by *Enterobacteriales*, *Bacteroidales*, *Coriobacteriales* and *Bifidobacteriales*. It was noted that addition of *L. gasseri* LM19 with *C. perfringens* gave a profile with more similarity to the control or *L. gasseri* LM19 only samples.

In *L. gasseri* LM19 treatment of donor 3 samples, *Bifidobacteriales* and *Enterobacteriales* increased over time in a similar way to the control, but *Clostridiales* were almost completely replaced by *Lactobacillales*. This rise was not as large when the *L. gasseri* LM19 was co-inoculated with *C. perfringens*, while addition of *C. perfringens* alone did not manage to maintain levels of *Clostridales*, with increases seen in *Enterobacteriales*, *Bifidobacteriales* and *Lactobacillales*. In this case, the *L. gasseri* LM19 with *C. perfringens* co-treatment at 24 h was more similar to the control, with the exception of the presence of *Lactobacillales*, suggesting that the *C. perfringens* effect on the microbial composition was changed by the inoculation with *L. gasseri*.

#### Presence of *L. gasseri* LM19 increases SCFA content in a colon model environment

Increases in the production of formic, acetic, propionic and butyric acids were observed in the three faecal fermentations in colon model conditions inoculated with *L. gasseri* LM9 alone compared to controls. However, there was a high variability in SCFA production between the three donors (Fig. 6d). In donor 1, production of SCFA, ethanol, succinate and, at 8 h only, lactate was increased compared to the control. In donor 2 there were notable increases in lactic acid from 4 h. Given the similar relative abundance of *Lactobacillales* (Fig. 6d) in control and *L. gasseri* treatment, this suggests an influence of *L. gasseri* LM19 on the native microbiota. SCFA content in vessels inoculated with *C. perfringens* indicated that this bacterium also had the capacity to increase SCFA levels, while values from the co-inoculated vessels indicated that SCFA synthesis from each bacterium was not notably affected by co-inoculation (data not shown).

## Discussion

In this study, we report the ability of a representative of the human breast milk microbiota to exhibit antagonistic activity against different enteropathogens and to produce both previously identified bacteriocins and one novel bacteriocin. We observed that different carbon sources have an influence on the expression of these bacteriocin genes. *L. gasseri* LM19 survived and expressed these antimicrobial genes in a complex faecal environment under simulated colon conditions. This can be considered an important feature, since not all strains that exhibit probiotic traits are able to survive in colon conditions and, therefore, deliver their activity in situ. Additionally, we observed other characteristics that are considered useful, such as the production of SCFA.

Gassericins are antimicrobial peptides produced by *L. gasseri*. Several gassericins have been identified as two-peptide class II bacteriocins, and some isolates have been shown to contain pairs of two-peptide operons. These two-peptide bacteriocins also show similarities with other two-peptide bacteriocins isolated from species previously grouped with *L. gasseri* (Tahara et al. 1996). The genome of *L. gasseri* LM19 encodes a helveticin J-like protein and two gene clusters of two-peptide bacteriocins, encoding peptides that show homology to acidocin LF221A and Gas K7A (Bact_1 and Bact_2) or to acidocin LF221B and Gas K7B (Gam A and GamX). Additionally, in the second half of one of these clusters, we observed the presence of structural genes encoding GamM and GamY, corresponding to a new two-component bacteriocin that showed a greater variation in sequence to previously described gassericins. Although these structural genes were co-located with several putative immunity genes, there were no separate genes for regulation or transport, which suggests that like Gam A and GamX they may use the *gamPKR* and *gamTC* located in the same cluster.

MALDI TOF MS analysis of cell extracts confirmed that masses for all 6 peptides were present. Further HPLC fractionation showed that the masses could be separated and associated with antimicrobial activity. However, yields of the single peptides in fractions varied, and the presence of traces of other peptides could not be ruled out, so a direct comparison between activities was problematic. Accordingly, synthetic peptides were produced and assayed. With both HPLC fractions and synthetic peptides, GamA and Bact_2 consistently gave the highest antimicrobial activity, but the activity of synthetic GamM was still notable. It is interesting that synthetic GamY did not show activity, while HPLC fractions containing the GamY mass did—this may be due to trace levels of other peptides in the fraction, which may either contribute their own activity or interact with GamY to facilitate its activity, or to a requirement for some further peptide processing which was not replicated by synthesis. Similarly, the activity for synthetic Bact_1 was lower than that from fractions containing the Bact_1 mass, presumably for the same reasons. Synergistic activity was reported previously between GasT and GatX and between the two components of gassericin S (here Bact_1 and Bact_2), respectively (Kasuga et al. 2019). It is possible that GamY requires the presence of one of the other peptides, possibly GamM, for activity. However, examination of possibly synergy between peptides did not identify any activity from GamY when next to GamM. There was very clear synergy between Bact_1 and Bact_2, and possible synergistic activity between GamA and Bact_1, but the GasT and GatX homologues from this study, GamA and GamX, did not appear to affect each other.

Due to the production of multiple bacteriocins, the activity of single peptides, peptide pairs or possible synergistic reactions are challenging to assess. The antagonistic effect on tested pathogens suggest that these bacteriocins may have different targets or modes of action, as both Gram-positive and Gram-negative bacteria are affected and the nature of the test had an impact on the sensitivity. Although the expression of genes for GamM and GamY was shown to increase notably with different carbon sources, the antimicrobial activity of the culture did not follow the pattern of *gamM* and *gamY* expression, indicating that the whole activity encompasses several if not all bacteriocins. Further analysis of a series of multigene knockouts which synthesise single peptides or pairs of peptides should give a clearer analysis of peptide activity and interaction.

The presence of bacteria in human breast milk has been reported previously and the existence of a bacterial entero-mammary pathway has recently been proposed (Rodríguez 2014). These bacteria might have a gut origin and that could explain their ability to survive in GI tract conditions and exhibit antagonistic traits against other gut bacteria, such as enteropathogens, that might share the same environment. This may also explain their ability to utilise complex carbohydrate polymers which are likely to be found in the gut environment, and the observed up-regulation of bacteriocin genes *gamM* and *gamY* in a low-sugar environment or with certain complex carbohydrates might produce a competitive advantage. In previous work it was demonstrated that another potentially probiotic *L. gasseri*, strain K7, which produced 2 two-peptide bacteriocins K7 A, K7 A (cf), K7 B and K7 B (cf), was able to survive in faecal samples. Its bacteriocins were also the focus of examination by conventional PCR and RT-PCR (Treven et al. 2013). In that instance, the authors noted that bacteriocin genes were amplified by PCR from other LAB species present in the environment. However, in our controls and treatments where *L. gasseri* LM19 was not present, no PCR products were detected.

*L. gasseri* LM19 showed mixed effects on a strain of *C. perfringens* added to faecal fermentations of three different donors, causing a slight decrease in *C. perfringens* in only 2 out of 3 fermentations. This might indicate that the surrounding microbiota plays a synergistic or antagonistic role on the effect of *L. gasseri* LM19. However, it should be noted that in antimicrobial assays *C. perfringens* was only inhibited by *L. gasseri* LM19 cells, not cell-free supernatant, which might suggest that they should be in close proximity for an antimicrobial effect. Co-inoculation of *L. gasseri* LM19 with *C. perfringens* did seem to alter the effect of *C. perfringens* on the background microbiota: in experiments with all three donors, the profiles seen after addition of *L. gasseri* LM19 with *C. perfringens* were more similar to instances where *L. gasseri* was added alone or in control samples than to samples containing only *C. perfringens*. Although we confirmed that bacteriocin genes were being expressed in these fermentations, other factors such as lactic acid production may also have an impact.

Colon model fermentations also allowed the production of formic, acetic, propionic and butyric acids to be quantified using NMR. We observed an increase in SCFA production in the faecal fermentation of the three donors when *L. gasseri* LM19 was added compared to the control. However, as with the microbial composition, the amount of each SCFA produced was very different from one donor to another, which might be related to production by other members of the microbiota that varied between the three donors. In a previous study of consumption of a beverage prepared with *L. gasseri* CP2305, the stools of the participants also presented an increased level of SCFA, while the microbiota experienced some alterations, including an increase in the presence of bacteria from *Clostridium* cluster IV, known for producing higher amounts of SCFA (Sawada et al. 2016). The authors of that study could not conclude if the increase of SCFA was due to the effect of *L. gasseri* or due to the proliferation of bacteria that produced more SCFA. Similarly, our addition of *C. perfringens* also increased SCFA levels to greater than those seen with *L. gasseri* LM19. SCFA production also depends on diet and availability of nutrients in the gastrointestinal tract as well as the resident microbiota (den Besten et al. 2013; Holmes et al. 2017).

This work shows the ability of *L. gasseri* LM19, a multi-bacteriocin breast milk isolate, to survive in colon conditions. Its ability to express different bacteriocin genes, including a novel gassericin M, under these conditions, makes it an interesting candidate for further studies.

## Electronic supplementary material

ESM 1 (PDF 891 kb)
