# Supplementary material for: Production of multiple bacteriocins, including the novel bacteriocin gassericin M, by Lactobacillus gasseri LM19, a strain isolated from human milk
Source: Appl Microbiol Biotechnol. 2020 Mar 13;104(9):3869–84. doi: 10.1007/s00253-020-10493-3 (PMC7162838; doi:10.1007/s00253-020-10493-3)
Supplement: Supplementary file 1 — (PDF 891 kb) [file 253_2020_10493_MOESM1_ESM.pdf]

Applied Microbiology and Biotechnology

Production of multiple bacteriocins, including the novel bacteriocin gassericin M, by *Lactobacillus gasseri* LM19, a strain isolated from human milk

Supplementary Information

Enriqueta Garcia-Gutierrez[1,2] 0000-0001-5683-7924
Paula M. O'Connor[2,3] 0000-0001-6462-2077
Ian J. Colquhoun[1]
Natalia M.Vior[4] 0000-0003-1890-3884
Juan Miguel Rodríguez[5]
Melinda J. Mayer[1] 0000-0002-8764-2836
Paul D. Cotter[2,3*] 0000-0002-5465-9068
Arjan Narbad[1]

[1] Gut Microbes and Health Institute Strategic Programme, Quadram Institute Bioscience, Norwich, UK

[2] Food Bioscience Department Teagasc Food Research Centre, Moorepark, Fermoy, Cork, Ireland

[3] APC Microbiome Ireland, University College Cork, Cork, Ireland

[4] Molecular Microbiology, John Innes Centre, Norwich, UK

[5] Dpt. Nutrition and Food Science, Complutense University of Madrid, Madrid, Spain

*Corresponding author:

Paul D. Cotter

paul.cotter@teagasc.ie

Tel. +353 (0)25 42694; +353 (0)76 1112694 (Lync)

Table S1 Relative gene expression levels of different bacteriocin genes from *L. gasseri* LM19 grown in MRS supplemented with different carbon sources.

		No supplemental carbon source	Glucose	Lactose	Galactose	Inulin	Starch	Pectin
Helveticin J-like	24 h	2.21	0.81	0.37	0.94	2.10	6.09	3.20
		2.98	0.94	0.89	0.87	2.13	8.23	2.33
		3.14	1.24	1.79	1.42	2.34	4.58	3.46
	48 h	1.53	1.11	2.12	0.92	3.16	2.61	4.68
		2.01	1.20	1.91	0.53	1.80	2.33	1.70
		1.78	0.67	2.51	1.07	0.79	2.45	3.55
gamA	24 h	3.18	0.75	1.01	1.64	0.11	1.37	0.21
		ND	0.57	1.06	2.26	0.08	0.71	0.38
		1.55	1.67	0.54	1.16	0.09	1.54	0.32
	48 h	0.87	1.03	0.63	1.05	0.31	0.13	0.39
		1.11	1.15	0.45	1.49	0.65	0.78	0.47
		0.47	0.81	0.59	0.88	0.39	ND	0.45
gamX	24 h	ND	0.80	0.44	0.43	0.21	0.70	0.39
		3.62	0.78	0.90	2.11	0.18	0.62	0.34
		1.86	1.40	0.52	1.34	0.13	2.21	0.33
	48 h	1.12	1.04	0.78	1.13	0.83	1.26	0.52
		1.05	1.33	0.58	0.93	1.15	0.80	0.45
		1.13	0.62	0.70	0.42	0.81	ND	0.51
bact_1	24 h	0.00	0.40	1.09	1.80	0.58	0.01	0.20
		0.00	0.97	0.81	1.69	0.80	0.03	0.29
		0.95	1.62	0.99	0.99	0.93	0.07	0.82
	48 h	0.03	1.09	1.15	1.97	0.82	0.00	0.80
		0.02	0.75	1.47	2.04	0.80	0.00	0.82
		0.02	1.15	1.09	1.01	0.98	0.00	0.69
bact_2	24 h	0.01	0.57	0.71	0.65	0.42	0.06	0.13
		ND	0.38	0.68	1.24	0.31	0.01	0.20
		0.29	2.04	1.34	1.20	0.69	0.04	0.68
	48 h	0.04	1.00	1.43	2.15	0.55	ND	0.94
		0.03	0.80	2.12	1.31	0.63	0.01	1.00
		0.03	1.19	1.78	0.77	0.59	0.00	0.05

Values are relative to the expression of each gene in glucose supplemented cultures after 24h incubation; ND, not detected

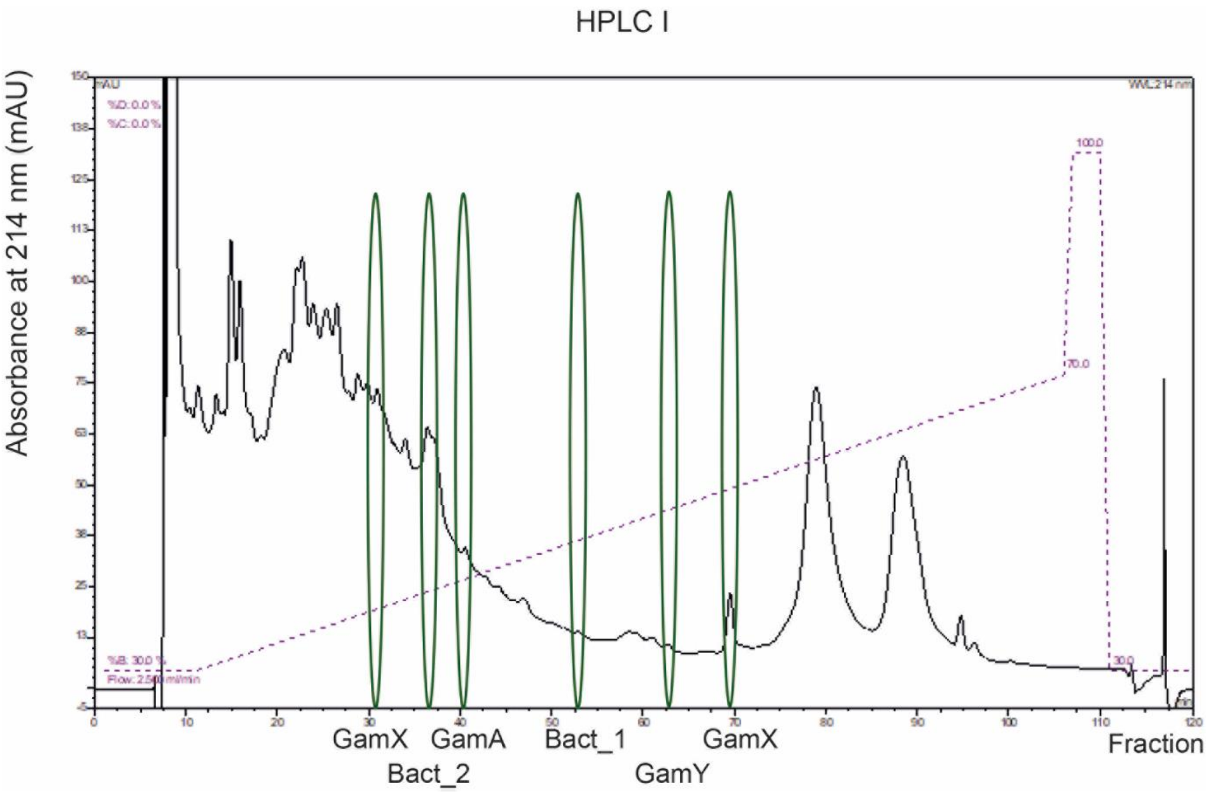

Fig S1 HPLC chromatogram showing fractionation I of *L. gasseri* LM19 cell extracts; MALDI TOF MS chromatograms of these fractions are shown in Figure S3. These fractions were purified further (see Fig. S2).

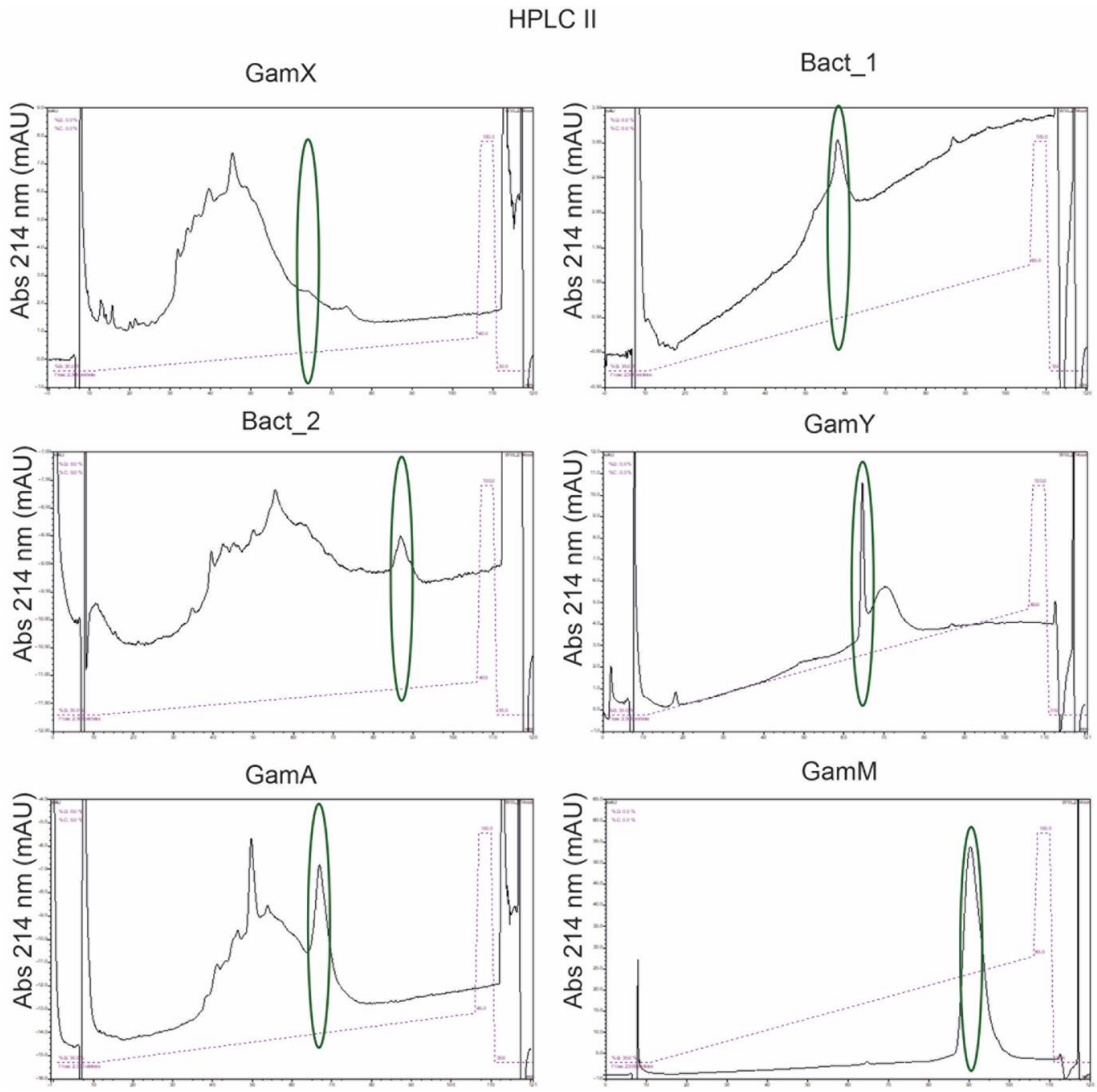

Fig S2 HPLC chromatograms from HPLC fractionation II of *L. gasseri* LM19 cell extracts fractions; absorbance peaks associated with putative bacteriocins (see titles) are encircled; MS chromatograms of these fractions are shown in Figure S3.

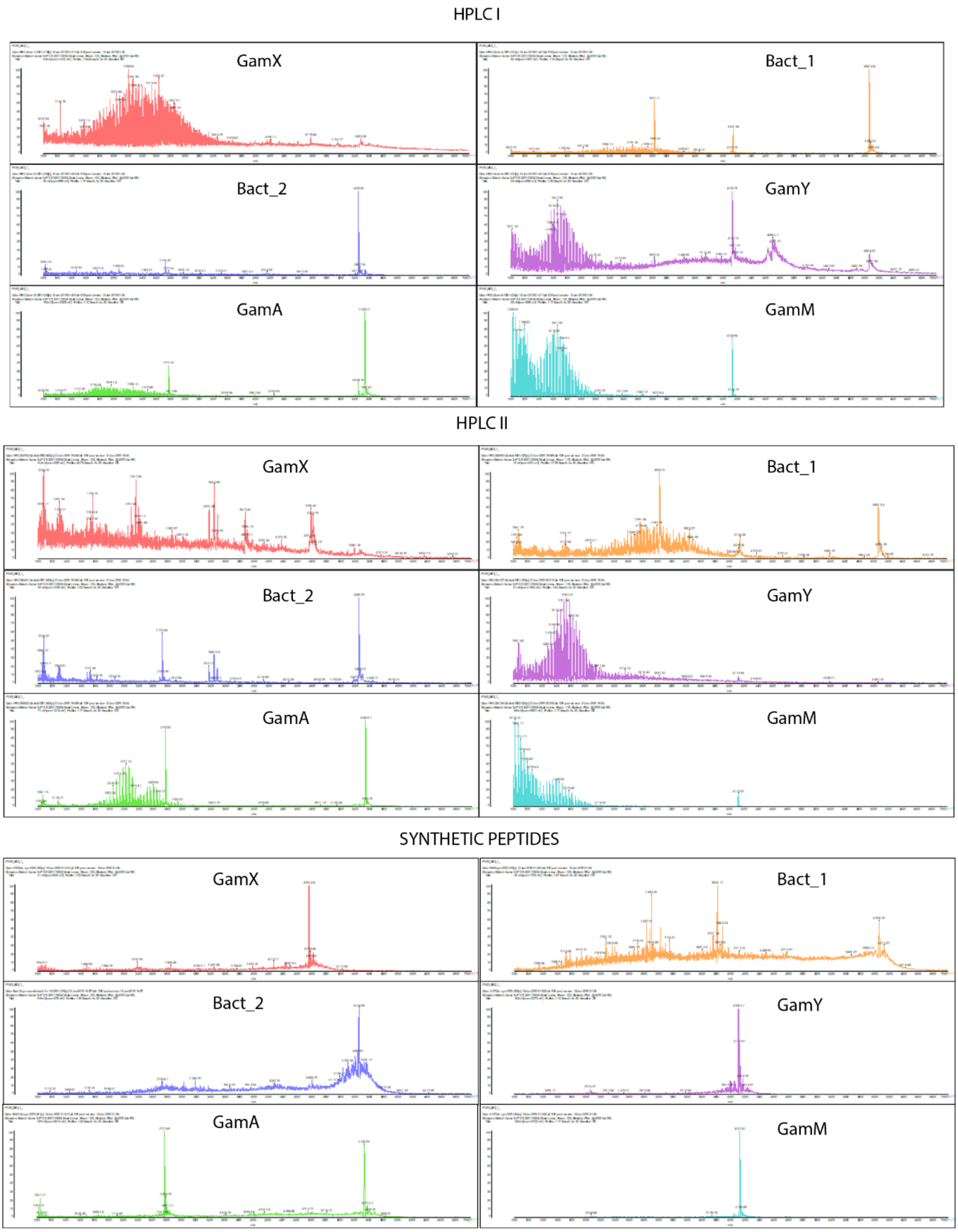

Fig S3 MALDI TOF MS chromatograms of cell extract fractions showing antimicrobial activity from HPLC fractionation I HPLC fractionation II, and synthetic peptides.
